# Intestinal fructose catabolism promotes obesity and insulin resistance via ileal lacteal remodeling

**DOI:** 10.1126/sciadv.aec0481

**Published:** 2026-08-28

**Authors:** Miranda L. Lopez, Taekyung Kang, Ana M. Espeleta, Varvara I. Rubtsova, Jongwon Baek, Jakob Songcuan, Elena M. Moyer, Joohwan Kim, Won-Suk Song, Sunhee Jung, Rochelle W. Lai, Aiyana Binuya, Christopher H. Lee, Amy R. Lantz, Nicholas D’Sa, Alexis Anica, Elise Tran, Yujin Chun, Wonsuk Choi, Ki-Hong Jang, Sungji Cho, Miranda E. Kelly, Ian J. Tamburini, Yasmine H. Alam, Johnny Le, Cuauhtemoc B. Ramirez, Raghu P. Kataru, Seon Pyo Hong, Thomas Q. de Aguiar Vallim, Dequina A. Nicholas, Katherine S. Xue, Gina Lee, Hosung Bae, Cholsoon Jang

**Affiliations:** ^1^ Department of Biological Chemistry, Center for Epigenetics and Metabolism, Chao Family Comprehensive Cancer Center, School of Medicine, University of California Irvine, Irvine, CA, USA.; ^2^ Department of Molecular Biology and Biochemistry, University of California Irvine, Irvine, CA, USA.; ^3^ Center for Complex Biological Systems, University of California, Irvine, Irvine, CA, USA.; ^4^ Department of Microbiology and Molecular Genetics, University of California Irvine School of Medicine, Irvine, CA, USA.; ^5^ Division of Cardiology, Department of Medicine, David Geffen School of Medicine, University of California, Los Angeles (UCLA), Los Angeles, CA, USA.; ^6^ Department of Surgery, Memorial Sloan Kettering Cancer Center, New York, NY, USA.; ^7^ Center for Vascular Research, Institute for Basic Science, Daejeon, Korea.; ^8^ Department of Ecology and Evolutionary Biology, University of California Irvine, Irvine, CA, USA.; ^9^ Division of Endocrinology and Metabolism, Department of Internal Medicine, University of Iowa, Iowa City, IA, USA.; ^10^ Department of Chemistry, School of Physical Sciences, University of California Irvine, Irvine, CA, USA.

## Abstract

High-fructose corn syrup (HFCS) consumption is a risk factor for obesity and diabetes, yet the underlying mechanisms, especially at the specific organ level, are incompletely understood. Catabolism of dietary fructose primarily occurs in the small intestine and liver, with fructose breakdown in the liver being pathological, while small intestinal fructose clearance protects the liver. Here, we report that inhibition of fructose catabolism specifically in the murine small intestine unexpectedly mitigates fructose-induced obesity and insulin resistance. Such phenotypes are attributed to decreased dietary fat absorption by the shortening of ileal lacteals. Fecal transplantation experiments revealed that the microbiome altered by blunted host intestinal fructose catabolism decreases ileal macrophages essential for lacteal growth. Thus, altered intestinal lacteal architecture likely contributes to the synergistic effects of high fat and sugar on metabolic disorders. It may also be relevant to the clinical evidence that pharmacologic suppression of fructose catabolism mitigates diet-induced obesity.

## INTRODUCTION

Over the past century, sugar has steadily crept into modern diets, a trend particularly prominent in North and South America (*1*, *2*). This trend is largely attributed to the food industry’s embrace of high-fructose corn syrup (HFCS) to enhance the flavor of almost all processed food products. While dietary fat used to be regarded as the leading culprit of public health, in recent years, researchers have found that the added sugars in the diet, mainly in the form of fructose, are primarily contributing to the prevalence of obesity, diabetes, and metabolic dysfunction–associated steatotic liver disease (MASLD) (*2*–*5*). Although fructose and glucose, the two principal dietary sugars, carry the same amount of calories, their impact on the body differs drastically due to their distinct metabolism. For example, compared to glucose, fructose acts as a potent inducer of de novo lipogenesis and inflammation, leading to hepatic steatosis, fibrosis, and increased adiposity across organs (*6*).

We and others previously demonstrated that fructose metabolism initiates in the small intestine (*7*–*10*), driven by the expression of Ketohexokinase-C (*Khk-C*), a highly active isoform of *Khk* that phosphorylates fructose to fructose-1-phosphate (*11*). Genetic deletion of *Khk-C* in the small intestine under 10% sucrose-drinking conditions causes fructose spillover to the liver, leading to excessive hepatic fructose catabolism and MASLD development (*8*). Conversely, individuals with essential fructosuria, a hereditary whole-body deficiency of *Khk*, rarely develop metabolic disorders not only because they avoid fructose intake due to fructose intolerance but also because ingested fructose is barely catabolized in the liver (*12*, *13*). Instead, dietary fructose in these individuals is metabolized in other tissues that express hexokinase such as muscle, kidneys and adipose tissues (*10*, *14*), with 10 to 20% being excreted in the urine (*15*, *16*).

Unlike highly regulated glycolytic enzymes, *Khk* is not regulated allosterically (*17*). Accordingly, fructose catabolism occurs immediately once the intestinal or hepatic cells encounter fructose. However, other host-specific factors than just Khk expression likely influence fructose catabolism and tissue responses. For example, the villus length of the small intestine, which can be increased by chronic consumption of a high-fat diet, may enhance fructose absorption and catabolism (*18*). However, how intestinal fructose catabolism affects conversely the intestinal architecture, gut immune landscape, and microbiome remains unknown.

Here, we found that blunting fructose catabolism in the small intestine protects mice against fructose-induced obesity and insulin resistance, which was associated with decreased ileal lacteal length and dietary fat absorption. The gut microbiome and villus-resident macrophages play critical roles in this process. These data reveal a previously unrecognized connection between intestinal fructose catabolism and dietary fat absorption through ileal lacteal development, providing important insights into understanding metabolic disorders caused by the high-fat/high-sugar diet consumption in modern society.

## RESULTS

### Impact of intestinal *Khk-C* deletion on body fitness and glucose homeostasis

**Fig. 1.**
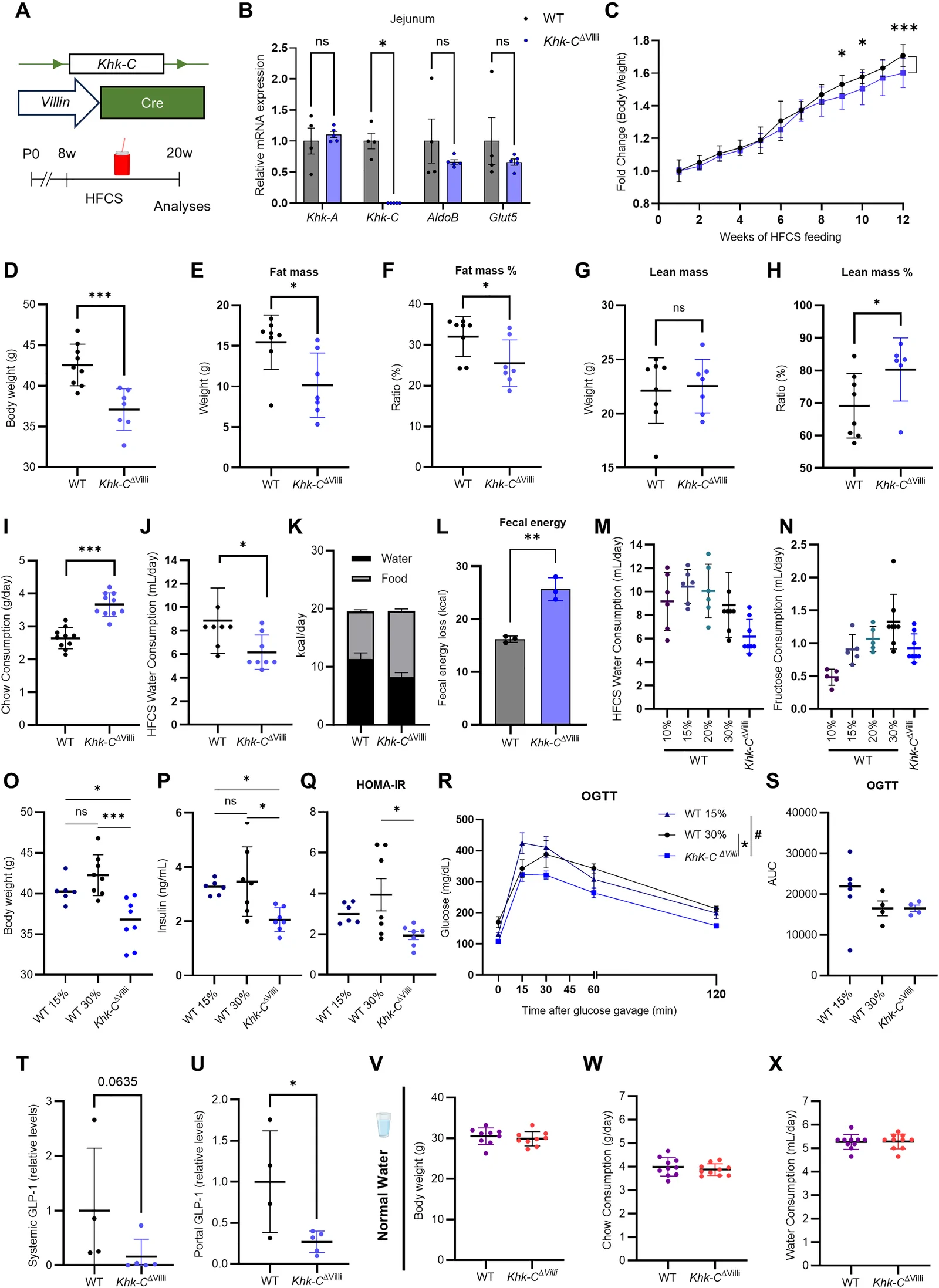
Suppression of intestinal fructose catabolism mitigates HFCS-induced obesity and insulin resistance. (**A**) Eight-week-old WT or *Khk-C*^∆*Villi*^ male mice were fed water containing 30% HFCS for 12 weeks. (**B**) Fructose catabolism gene expression in jejunum after 30% HFCS intake for 12 weeks (w; *n*_WT_ = 4, *n*_Khk-C_ = 5). (**C** and **D**) Body weight changes and final body weight of mice on HFCS (*n*_WT_ = 9, *n*_Khk-C_ = 7). (**E** and **F**) Fat mass (E) and the ratio of fat mass to body weight (%) (F) (*n*_WT_ = 6, *n*_Khk-C_ = 4). (**G** and **H**) Lean mass (G) and the ratio of lean mass to body weight (%) (H) (*n*_WT_ = 6, *n*_Khk-C_ = 4). (**I** to **K**) Daily chow consumption (I), water intake (J), and total calorie intake (K) per mouse on HFCS (*n* = 10). (**L**) Fecal energy loss (*n* = 3). (**M** and **N**) Daily water intake (M) and total fructose consumption (N) per mouse on varying concentrations of HFCS (*n*_WT10-20%_ = 6, *n*_WT30%, Khk-C_ = 8). (**O**) Final body weight of mice on 15% or 30% of HFCS (*n*_WT15%_ = 6, *n*_WT30%, Khk-C_ = 8). (**P** to **S**) Fasting insulin (P), homeostatic model assessment of insulin resistance (HOMA-IR) (Q), oral glucose tolerance test (OGTT) (R), and area-under-the-curve (AUC) (S) of mice on 15 or 30% of HFCS (*n*_WT15%_ = 6, *n*_WT30%, Khk-C_ = 4). (**T** and **U**) Systemic and portal blood levels of GLP-1 (*n*_WT_ = 4, *n*_Khk-C_ = 5). (**V**) Final body weight of mice fed normal water (NW) without HFCS (*n* = 9). (**W** and **X**) Daily chow (W) or water (X) consumption per mouse on NW (*n* = 10). Data are mean ± SD. ns, not significant. *P < 0.05, **P < 0.01, and ***P < 0.001 by one-way ANOVA followed by Tukey’s test (O to S) or Student’s *t* test. ^#^*P* < 0.05 and ^###^*P* < 0.001 by repeated-measures ANOVA (C and R).

To study the role of small intestinal fructose catabolism in metabolic pathophysiology, we used intestine-specific *Khk-C* deletion mice (*Khk-C*^∆Villi^) (*8*), which have *lox*P sites around a *Khk-C*-specific exon and intestine-specific Cre (*Villin*-Cre) (Fig. 1A). These mice exhibit intact *Khk-A* expression and a trend of decrease in other fructose-specific genes, such as *Aldolase B* (*AldoB*) and *Glucose transporter 5* (*Glut5*) (Fig. 1B and figs. S1, A and B). When exposed to a relatively low-dose fructose regimen (10% sucrose in drinking water), these mice exhibited increased hepatic lipogenesis and steatosis (*8*, *10*). However, because mice are much more resistant to fructose than humans, investigators used a high-dose (30%) HFCS 55, composed of 55% fructose and 45% glucose (*11*, *19*–*21*) to study the pathogenic impact of fructose observed in humans. Accordingly, we also adopted this regimen in this study.

After being fed 30% HFCS in their drinking water for 12 weeks, *Khk-C*^∆Villi^ mice gained less weight than littermate controls [wild type (WT)] (Fig. 1, C and D). This phenotype was attributed to lower fat mass in *Khk-C*^∆Villi^ mice (Fig. 1, E and F), whereas lean mass trended toward being higher (Fig. 1, G and H). *Khk-C*^∆Villi^ mice showed higher chow consumption and lower water consumption than WT mice (Fig. 1, I and J). These changes balanced out, leading to no difference in total caloric intake (Fig. 1K). To further investigate the reduced body weight despite similar caloric intake, we quantified total fecal energy output by oxygen bomb calorimetry and fructose excretion by liquid chromatography–mass spectrometry. Khk-C^∆Villi^ mice exhibited increased fecal energy excretion (Fig. 1L) with no detectable fructose excretion (fig. S1C), suggesting that enhanced fecal energy loss contributes, at least in part, to their lower body weight. To mitigate the influence of different fructose consumption on weight gain, we tested varying doses (10, 15, and 20%) of HFCS water in WT mice to match 30% HFCS water consumption in *Khk-C*^∆Villi^ mice (Fig. 1M). We found that providing 15% HFCS water to WT mice best matched the fructose intake of *Khk-C*^∆Villi^ mice fed with 30% HFCS (Fig. 1N).

Even with the matched fructose consumption, *Khk-C*^∆Villi^ mice still gained less weight than WT during the 12-week feeding period (Fig. 1O). Furthermore, *Khk-C*^∆Villi^ mice showed a statistically significant reduction in fasting insulin levels (Fig. 1P), with no difference in fasting glucose levels (fig. S1D). *Khk-C*^∆Villi^ mice exhibited a lower trend of homeostatic model assessment of insulin resistance (HOMA-IR) and better glucose tolerance compared to WT mice (Fig. 1, Q and R). Area-under-the-curve analysis indicated that baseline differences primarily contributed to the differences between groups (Fig. 1, R and S). These systemic metabolic improvements were accompanied by enhanced hepatic insulin sensitivity, reduced expression of de novo lipogenesis genes, and lower triglyceride (TG) content and lipid accumulation (fig. S1, E to I). Thus, despite fructose spillover to the liver in the absence of intestinal fructose catabolism, the decreased body weight and energy loss likely promote hepatic and systemic insulin sensitivity and overall metabolic health under high-dose HFCS intake.

To examine whether hormonal changes drive the observed metabolic improvements in *Khk-C*^∆Villi^ mice, we conducted a Luminex-based quantitative screen for a panel of incretins, adipokines and pancreas-derived hormones (fig. S1, J and K). In *Khk-C^∆Villi^* mice only glucagon-like peptide 1 (GLP-1), the incretin that inhibits appetite (*22*), revealed a statistically significant decrease, especially in portal blood (Fig. 1, T and U). Despite the lower GLP-1 levels, *Khk-C*^∆Villi^ mice showed reduced weight gain, suggesting that the phenotype is not associated with GLP-1–mediated appetite control.

Recent studies have shown that fructose is endogenously synthesized from glucose through the sorbitol pathway and it plays some roles in metabolic dysfunctions (*23*, *24*). Supporting this notion of endogenously produced fructose, the small intestine expresses *Khk-C* basally even in the absence of dietary fructose intake (*25*). We thus assessed the impact of intestinal *Khk-C* ablation on weight gain under the conditions where dietary fructose was completely absent. However, in contrast to high-dose HFCS-feeding conditions, *Khk-C*^∆Villi^ mice fed normal water (NW) showed no differences in weight gain compared to WT mice (Fig. 1V). Food and water intake were also indistinguishable between the groups (Fig. 1, W and X). Thus, intestinal *Khk-C* deficiency improves body fitness and glucose homeostasis only under the conditions of high-dose HFCS intake.

### Impact of intestinal *Khk-C* deletion on dietary fat absorption

**Fig. 2.**
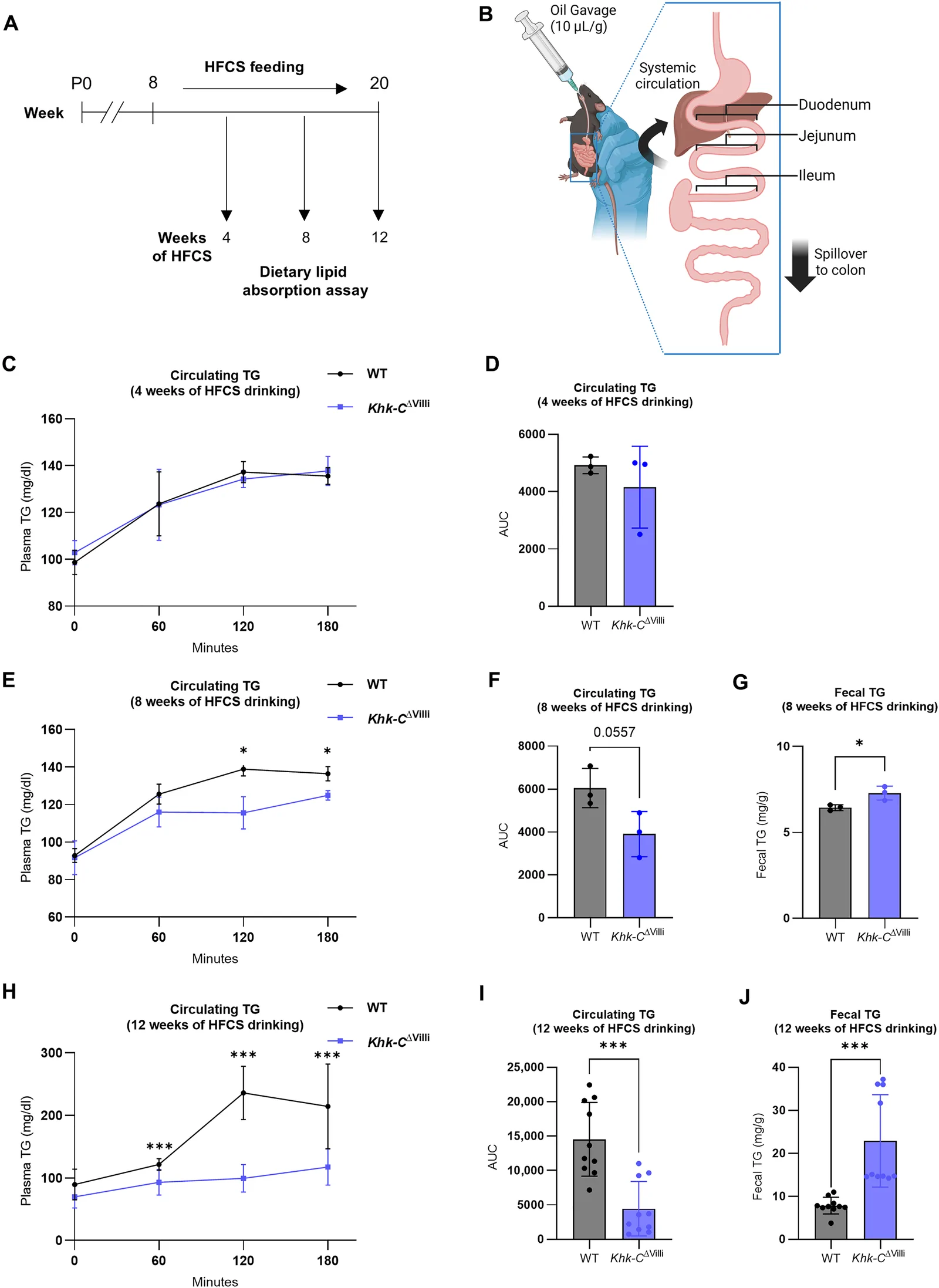
Suppression of intestinal fructose catabolism reduces dietary fat absorption under high-dose HFCS consumption. (**A** and **B**) During 12-week HFCS feeding, dietary fat absorption assay was performed on weeks 4, 8, and 12, using oral soybean oil provision followed by circulating and fecal triglyceride (TG) measurements. Figure partially created in BioRender. Jang, C. (2026) https://BioRender.com/9l87pmx. (**C** and **D**) Measurements of circulating TG levels (C) and the AUC (D) after 4 weeks of HFCS drinking (*n* = 3). (**E** to **G**) Measurements of circulating TG levels (E) and the AUC (F), and fecal TG excretion (G) after 8 weeks of HFCS drinking (*n* = 3). (**H** and **I**) Measurements of circulating TG levels (H) and the corresponding AUC (I) after 12 weeks of HFCS drinking (*n*_WT_ = 10, *n*_Khk-C_ = 10). (**J**) Measurements of TG excretion in feces collected 120 min post-oil gavage after 12 weeks of HFCS drinking (*n*_WT_ = 10, *n*_Khk-C_ = 10). Data are mean ± SD. *P < 0.05, ***P < 0.001 by Student’s *t* test.

We next investigated the underlying mechanism. Given that *Khk-C*^∆Villi^ mice showed lower fat mass (Fig. 1, E and F) and higher fecal energy (Fig. 1L) despite higher food consumption (Fig. 1I), we speculated that the absorption of dietary fat may be negatively affected in *Khk-C*^∆Villi^ mice. To test this hypothesis, we fed *Khk-C*^∆Villi^ and WT mice HFCS for 12 weeks again (Fig. 2A), during which we conducted a dietary fat absorption assay through oral administration of soybean oil, followed by the measurement of circulating TG spikes (Fig. 2B). After feeding 4 weeks of HFCS, we did not observe any difference in circulating TG spikes (Fig. 2, C and D). Notably, however, after 8 weeks of HFCS consumption, *Khk-C*^∆Villi^ mice exhibited a reduction in circulating TG spikes (Fig. 2, E and F) while higher fecal TG content (Fig. 2G), suggesting defective dietary fat absorption. Coincidentally, this 8-week time point was around when *Khk-C*^∆Villi^ mice started to show less weight gain than WT mice (Fig. 1C). After 12 weeks of HFCS feeding, *Khk-C*^∆Villi^ mice displayed substantially lower circulating TG spikes (Fig. 2, H and I) and higher fecal TG content (Fig. 2J) than WT mice. Thus, we concluded that ablating intestinal fructose catabolism during high-dose HFCS consumption impairs dietary fat absorption.

### Impact of intestinal *Khk-C* deletion on lacteal growth

Dietary fat absorption is initiated by the uptake of fatty acids (after hydrolysis from lipids) into enterocytes via various transport proteins (Cd36, Fatp1, Fatp4, and Fabp2), followed by reassembly into TGs (*26*). Such TGs are then transported through intestinal lacteals, the unique lymphatic vessels within intestinal villi (*27*). We first measured the mRNA expression of various fatty acid transport proteins across the duodenum, jejunum, and ileum but found no notable reduction in *Khk-C*^∆Villi^ mice (fig. S2, A to C).

**Fig. 3.**
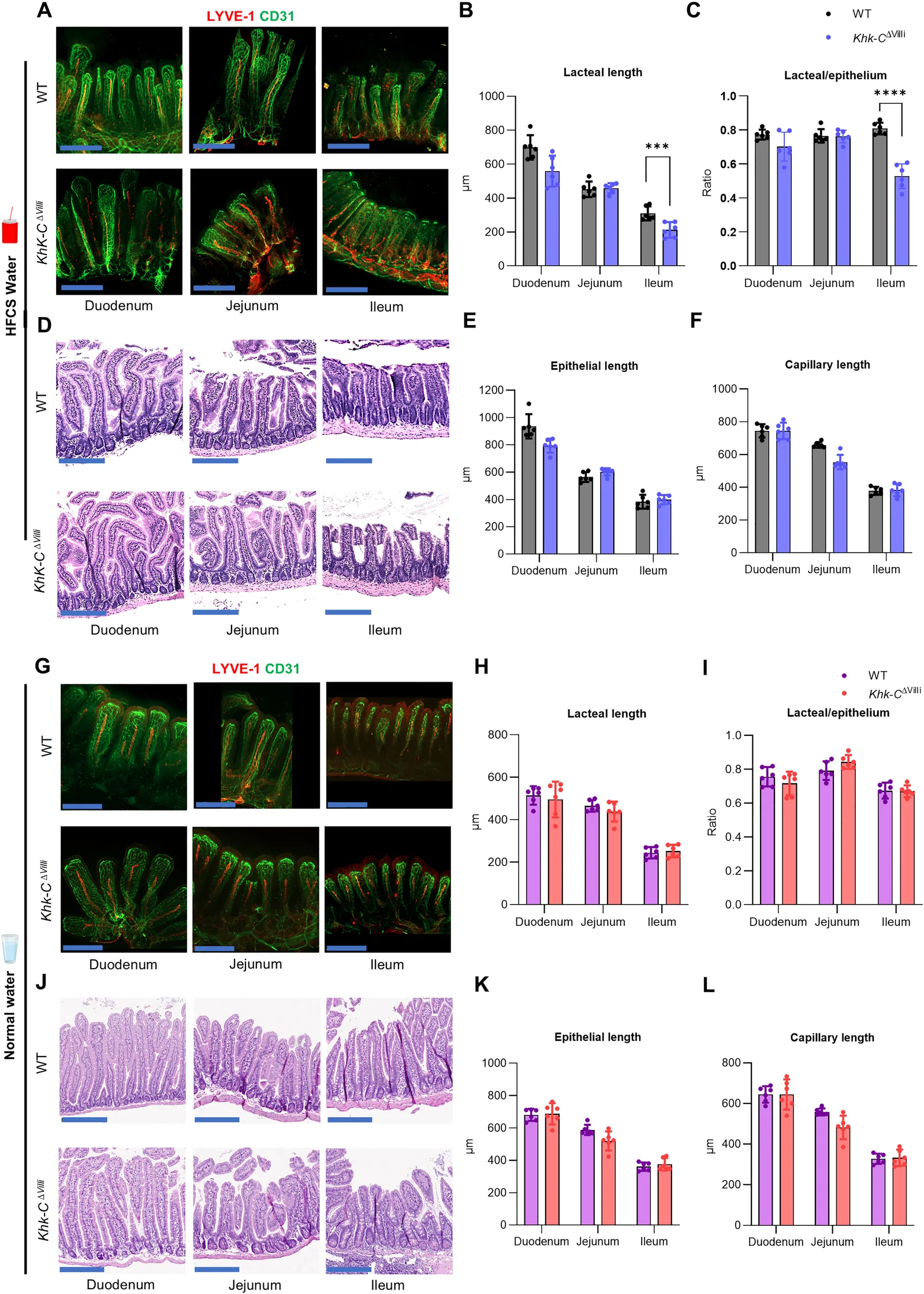
Suppression of intestinal fructose catabolism causes lacteal shortening under HFCS consumption. (**A**) Representative immunofluorescence images of intestinal whole mounts showing LYVE-1-positive lacteals (red) and CD31-positive capillaries (green) in mice on HFCS feeding for 12 weeks. Scale bars, 200 μm. (**B** and **C**) Lacteal length (B) and the ratio of lacteal/epithelial length (C) in intestinal tissues of mice on HFCS feeding (*n*_WT_ = 6, *n*_Khk-C_ = 6). (**D**) Representative H&E images of intestinal tissues of mice on HFCS feeding. Scale bars, 200 μm. (**E** and **F**) Epithelial (E) and capillary length (F) of intestinal tissues in mice on HFCS feeding (*n*_WT_ = 6, *n*_Khk-C_ = 6). (**G**) Representative immunofluorescence images of intestinal whole mounts showing LYVE-1–positive lacteals (red) and CD31-positive capillaries (green) in mice on NW feeding for 12 weeks. Scale bars, 200 μm. (**H** and **I**) Lacteal length (H) and the ratio of lacteal/epithelial length (I) in intestinal tissues of mice on NW feeding (*n* = 6). (**J**) Representative H&E images of intestinal tissues of mice on NW feeding. Scale bars, 200 μm. (**K** and **L**) Epithelial (K) and capillary length (L) of intestinal tissues in mice on NW feeding (*n* = 6). Data are mean ± SD. ns, not significant. ***P < 0.001 and ****P < 0.0001 by Student’s *t* test.

We then assessed the lacteals, whose architectures, including the length and surface area, are critical for lipid absorption (*28*, *29*). Whole-mount immunostaining of LYVE-1–positive lacteals revealed similar length between *Khk-C*^∆Villi^ and WT mice in the duodenum and jejunum (Fig. 3, A and B). In contrast, the ileum of *Khk-C*^∆Villi^ mice had shorter lacteals than that of WT mice (Fig. 3, A and B). The ratio of lacteal to epithelium was also lower in the ileum of *Khk-C*^∆Villi^ mice (Fig. 3C). Hematoxylin and eosin (H&E) staining indicated that the epithelial or capillary length was indistinguishable between *Khk-C*^∆Villi^ and WT mice (Fig. 3, D to F), indicating the shortening effect is specific to lacteals.

We next investigated whether the truncated lacteal in the ileum of *Khk-C*^∆Villi^ mice is also observed in the absence of dietary HFCS consumption. However, under NW drinking conditions, intestinal lacteals showed no reduction in their length in *Khk-C*^∆Villi^ mice (Fig. 3, G to I). The epithelial or capillary length was also not affected in NW drinking conditions (Fig. 3, J to L). Thus, both blunted intestinal fructose catabolism and dietary HFCS intake are required to trigger ileal lacteal shortening.

### The role of microbiome in the ileal lacteal growth and dietary fat absorption

**Fig. 4.**
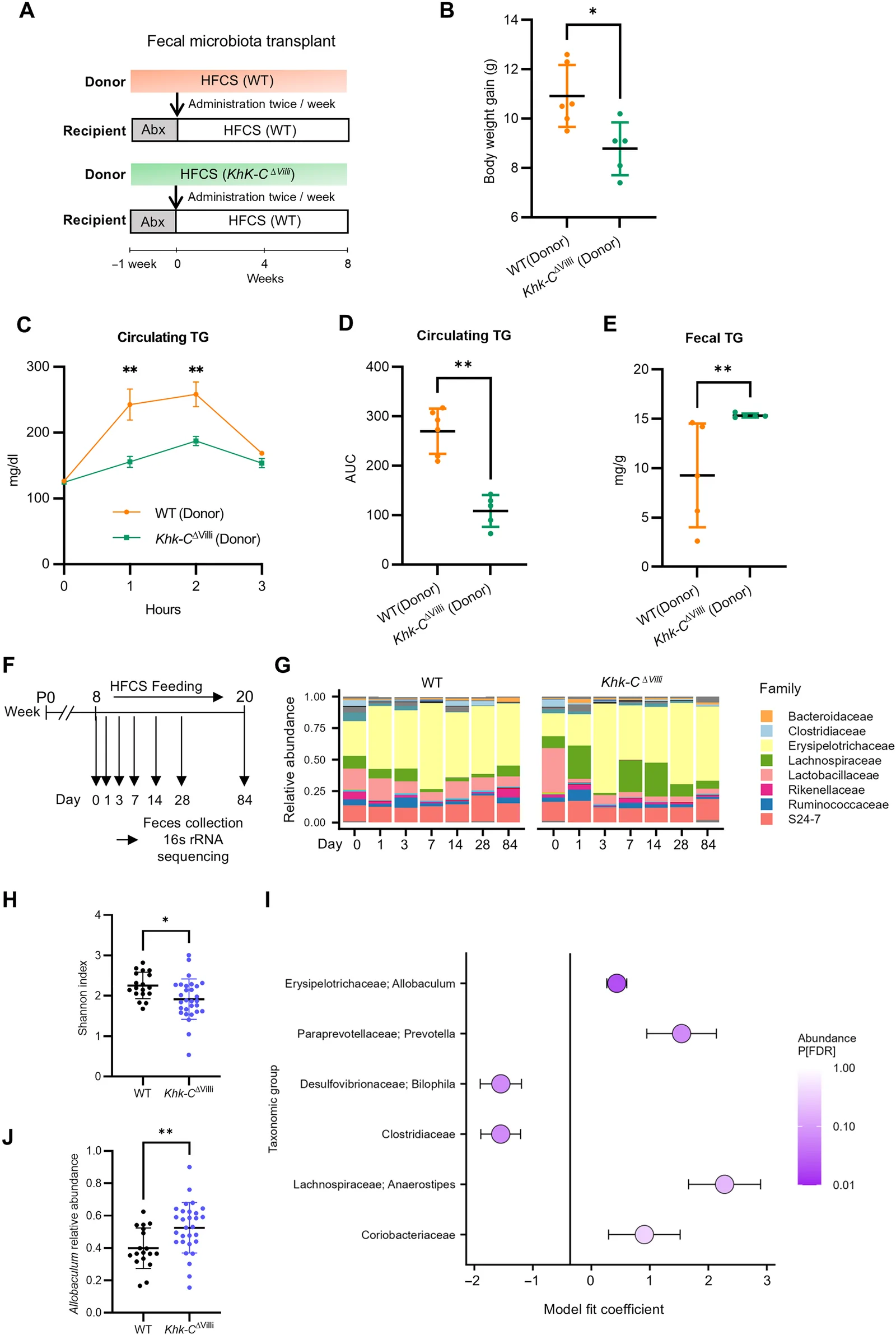
Fecal microbiome transplant phenocopies intestine-specific *Khk-C* depletion, which alters gut microbiome diversity under HFCS consumption. (**A**) Schematic of fecal transplant (twice/week) from donors (WT or *Khk-C* ^∆*Villi*^ mice on HFCS for 12 weeks; *n* = 6) to recipients (WT after antibiotics) with HFCS drinking for 8 weeks. (**B**) Body weight gain of the recipient mice after 8 weeks of fecal transplant and HFCS drinking (*n*_WT(Donor)_ = 6, *n*_Khk-C(Donor)_ = 5). (**C** to **E**) Measurements of circulating TG levels (C), their AUC (D), and fecal TG excretion (E) in the recipient mice following soybean oil gavage (*n*_WT(Donor)_ = 6, *n*_Khk-C(Donor)_ = 5). (**F**) Schematic overview of fecal 16*S* rRNA sequencing across multiple days of HFCS intake. (**G**) Representative bacterial family compositions from a WT and *Khk-C ^∆Villi^* mouse at each timepoint. (**H**) Alpha diversity (Shannon index) across all samples for WT and *Khk-C ^∆Villi^* mice post-day 14 of HFCS intake (*n*_WT_ = 18, *n*_Khk-C_ = 28). (**I**) Differential abundance results from MaAsLin3 showing the five taxa with the strongest associations between WT and *Khk-C ^∆Villi^* mice post-day 14 of HFCS intake *n*_WT_ = 18, *n*_Khk-C_ = 28). *X* axis represents the model fit coefficient (β) from MaAsLin3, indicating the direction and magnitude of association with the *Khk-C ^∆Villi^* genotype (positive = enriched in *Khk-C ^∆Villi^*, negative = enriched in WT). Only *Allobaculum* spp. was significant with a false discovery rate < 0.05. (**J**) Relative abundance of Allobaculum in WT and *Khk-C^∆Villi^* mice post-day 14 of HFCS intake (*n*_WT_ = 18, *n*_Khk-C_ = 28). Data are mean ± SD. *P < 0.05 and **P < 0.01 by Student’s *t* test.

We next sought to identify the mechanism underlying the shortened ileal lacteals in *Khk-C*^∆Villi^ mice. Previous research has identified gut microbiome as a crucial regulator of lacteal growth (*28*, *30*). We thus hypothesized that, blunted host intestinal fructose catabolism enhances gut microbiome exposure to dietary fructose, which leads to changes in their composition, affecting the lacteal growth. To test this idea, we conducted fecal transplant experiments. Specifically, we generated two groups of fecal donor mice: *Khk-C*^∆Villi^ and WT mice fed 30% HFCS for 12 weeks (Fig. 4A). In parallel, the recipient WT mice were first treated with a cocktail of antibiotics for a week to eliminate the gut microbiome. Then, we orally transplanted freshly isolated fecal contents from the donor mice to the recipient mice twice a week while feeding the recipient mice 30% HFCS for 8 weeks.

Notably, the mice that received fecal contents from *Khk-C*^∆Villi^ donors gained less weight during the 8-week feeding period than the mice that received fecal contents from WT donors (Fig. 4B). Consistent with their lower body weight, they also showed a trend of lower fasting glucose, insulin and HOMA-IR (fig. S3, A to C). We next compared dietary fat absorption between the recipient mouse groups. Oral fat absorption assay revealed substantially lower circulating TG spikes and more fecal TG excretion in the mice that received fecal contents from *Khk-C*^∆Villi^ donors than that in the mice that received fecal contents from WT mice (Fig. 4, C to E).

Next, to determine whether antibiotics pretreatment and HFCS consumption are required for successful microbiome colonization and maintenance (by continuous supply of HFCS from diet) to recapitulate the phenotype, we performed fecal transplant experiments using the same donors (*Khk-C*^∆Villi^ and WT mice fed 30% HFCS for 12 weeks), but different recipient mice that were neither pretreated with antibiotics nor exposed to 30% HFCS (fig. S4A). Under these conditions, fecal transplant did not affect body weights, glucose tolerance, and dietary fat absorption (fig. S4, B to F), indicating that prior microbiome depletion and sustained HFCS exposure are required to exert the effects.

We were curious whether changes in microbiome composition contribute to HFCS-induced weight gain and whether this effect is reversible by removing HFCS from the diet. To this end, WT mice were maintained on HFCS, NW, or switched from HFCS to NW over a 16-week feeding period (fig. S5A). Removal of HFCS from diet restored body weight, fat, and lean mass (fig. S5, B to H). However, microbiome composition changes were modest, as we did not observe substantial effects of dietary reversal on Jensen-Shannon divergence, although there were some differences in microbiome taxa enrichment between the groups (fig. S5, I to K). These data suggest that, at least in the presence of intact intestinal fructose catabolism, HFCS-induced weight gain is reversible, with a modest impact on gut microbiome.

These findings led us to investigate how gut microbiome composition was affected by HFCS intake in the absence of intestinal fructose catabolism. We thus collected fresh feces from WT and *Khk-C*^∆Villi^ mice during a 12-week HFCS feeding period and conducted 16*S* rRNA sequencing at multiple time points, 0, 1, 3, 7, 14, 28, and 84 days, to capture their dynamic changes (Fig. 4F and table S1). At baseline before HFCS provision (day 0), we did not observe a profound basal difference in microbiome composition between WT and *Khk-C*^∆Villi^ mice (table S1). Following HFCS feeding, we observed a statistically significant decrease in microbiome diversity in *Khk-C*^∆Villi^ mice (Fig. 4, G and H). MaAsLin3 differential abundance analysis revealed that some microbiome species, including *Allobaculum* spp. (family Erysipelotrichaceae), became more abundant in *Khk-C*^∆Villi^ mice than WT mice after HFCS consumption (Fig. 4, I and J). This finding was intriguing because prior studies have shown that members of the Erysipelotrichaceae family expand in mice on high-fat/high-sugar diets and affect the host’s lipid metabolism (*31*, *32*). While more investigations are needed to identify specific bacteria species that mediate fecal transplant effects, these data suggest that blunted host intestinal fructose catabolism can reshape the gut microbiome under high-dose HFCS consumption, which may, in turn, influence the host intestinal architecture.

### Impact of intestinal fructose catabolism on villus macrophages

**Fig. 5.**
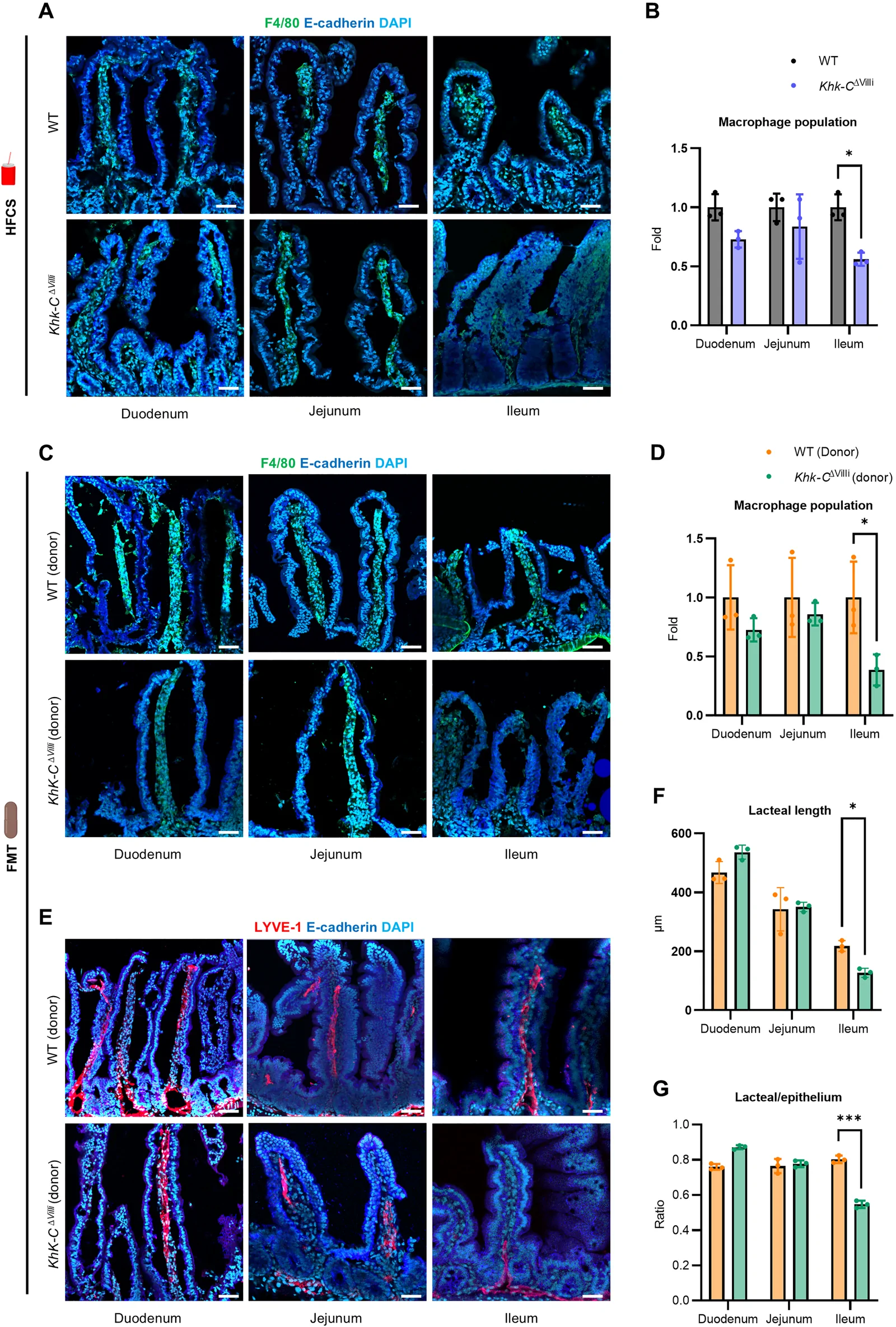
Suppression of intestinal fructose catabolism reduces villus macrophages under HFCS consumption and is phenocopied by fecal microbiome transplantation. (**A**) Representative immunofluorescence images of intestinal cryosections showing F4/80-positive macrophages (green), E-cadherin–positive epithelial cells (blue), and nuclei (DAPI, turquoise). Scale bars, 50 μm. (**B**) Relative abundance of macrophages in mice on HFCS for 12 weeks (*n* = 3). (**C**) Representative immunofluorescence images of intestinal cryosections showing F4/80-positive macrophages (green), E-cadherin–positive epithelial cells (blue), and nuclei (DAPI, turquoise) in the recipient mice after 8 weeks of fecal transplant and HFCS drinking. Scale bars, 50 μm. (**D**) Relative abundance of macrophages in the recipient mice (*n* = 3). (**E**) Representative immunofluorescence images of cryosections showing LYVE-1–positive lacteals (red), E-cadherin–positive epithelial cells (blue), and nuclei (DAPI, light blue) in the recipient mice after 8 weeks of fecal transplant and HFCS drinking. Scale bars, 50 μm. (**F** and **G**) Lacteal length (F) and the ratio of lacteal/epithelial length (G) in intestinal tissues of the recipient mice after 8 weeks of fecal transplant and HFCS drinking (*n* = 3). Data are mean ± SD. *P < 0.05 and ***P < 0.001 by Student’s *t* test.

We next investigated how the gut microbiome in *Khk-C*^∆Villi^ mice affects the lacteal growth and dietary lipid absorption. Considering that microbiome directly influences the intestinal immune system (*33*, *34*) and that villus-resident macrophages are critical sources of growth factors required for lacteal growth (*28*, *35*), we postulated that the gut microbiome of *Khk-C*^∆Villi^ mice cause lacteal shortening by affecting villus-resident macrophages. To test this hypothesis, we performed whole-mount immunostaining of the intestinal villi using the F4/80 antibody that marks macrophages. Consistent with the literature (*36*), F4/80-positive macrophages align along the lacteal (Fig. 5A). In the duodenum or jejunum, we found no change in the number of macrophages in *Khk-C*^∆Villi^ mice compared to WT mice (Fig. 5, A and B). In contrast, there were fewer (∼50%) macrophages in the ileum of *Khk-C*^∆Villi^ mice compared to WT mice (Fig. 5, A and B). This finding was consistent with the lacteal shortening only observed in the ileum of *Khk-C*^∆Villi^ mice (Fig. 3, A to C). Notably, *Khk-C*^∆Villi^ mice fed NW without dietary fructose did not show changes in the number of F4/80-positive macrophages (fig. S6, A and B), again indicating that HFCS intake is necessary to affect lacteal length in *Khk-C*^∆Villi^ mice.

To determine whether the altered gut microbiome in *Khk-C*^∆Villi^ mice is necessary or sufficient to decrease villus-resident macrophages and shorten ileal lacteals, we treated antibiotics to eradicate the gut microbiome in *Khk-C*^∆Villi^ mice or performed fecal transplant experiments. Treatment of an antibiotic cocktail before HFCS feeding (fig. S7A) did not result in further reductions in villus macrophage accumulation or lacteal length in *Khk-C*^∆Villi^ mice (fig. S7, B and C), suggesting that deficiency in certain microbiota species, induced by the lack of small intestinal fructose clearance and HFCS drinking, may contribute to the observed phenotype, and antibiotic treatment mimics this effect. This result was consistent with the previous studies that showed lacteal regression by the gut microbiota depletion (*28*, *37*). Next, we performed the fecal transplant experiments as previously (Fig. 4A). We found that WT mice receiving fecal transplants from *Khk-C*^∆Villi^ donors displayed a decrease in the number of villus macrophages in the ileum (Fig. 5, C and D). Furthermore, they exhibited a reduction in ileal lacteal length (Fig. 5, E and F) and a decreased ratio of lacteal to epithelial length in the ileum (Fig. 5G). There was no difference in epithelial length across different intestinal segments (fig. S6, C and D).

**Fig. 6.**
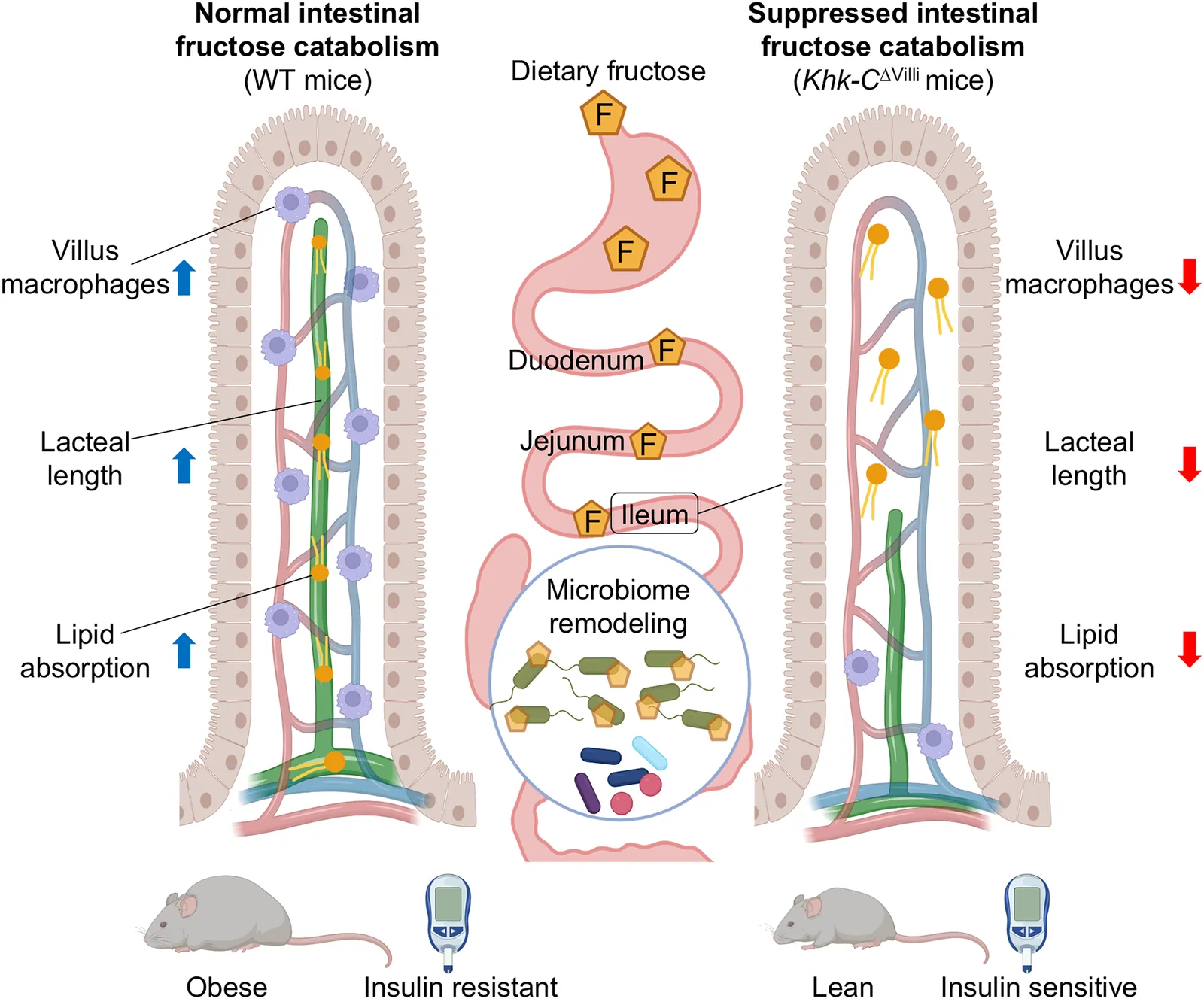
Catabolism of dietary fructose in the small intestine promotes obesity and insulin resistance by inducing microbiome-mediated ileal lacteal remodeling. In WT mice, dietary fructose is catabolized in the small intestine, altering the gut microbiome to promote villus macrophages that support ileal lacteal growth. This expansion of lacteal surface area enhances dietary lipid absorption, thereby contributing to obesity and insulin resistance. In contrast, in *Khk-C^∆Villi^* mice, inhibition of small intestinal fructose catabolism remodels the microbiome and reduces villus macrophages essential for lacteal growth. The resulting decrease in lacteal surface area limits lipid absorption and protects against obesity-associated insulin resistance. Figure partially created in BioRender. Jang, C. (2026) https://BioRender.com/zcnudyg.

Collectively, these findings underscore the importance of gut microbiome in mediating the impact of defective intestinal fructose catabolism on ileal macrophage remodeling and lacteal growth under high-dose HFCS conditions, which ultimately affects dietary fat absorption, weight gain, and insulin sensitivity (Fig. 6). This previously unappreciated link between intestinal fructose catabolism and dietary lipid absorption may explain the synergistically stronger effects of a diet enriched with both fat and fructose on deteriorating metabolic health compared to a diet enriched with either fat or fructose alone.

## DISCUSSION

In a contemporary society where excessive fructose and calories are widespread in processed foods, the small intestine’s role in absorbing and processing dietary nutrients becomes even more critical in determining the trajectory between health and disease. While many studies have investigated the impact of high-fat diet consumption on the absorption of other nutrients (*18*, *35*, *36*), how fructose consumption or organs’ fructose catabolic capacity influences the absorption of other nutrients is poorly understood. Here, we found an unexpected role of small intestinal fructose catabolism in modulating gut microbiome, ileum-specific lacteal growth, dietary fat absorption, and eventually whole-body metabolic fitness following the consumption of high-dose HFCS.

Individuals having loss-of-function mutations in *Khk* have been known to be resistant to fructose-associated pathologies, including obesity, diabetes, and MASLD (*12*, *13*). Without fructose consumption, such individuals have no overt phenotypes, consistent with our data in mice fed NW without dietary fructose. Accordingly, several *Khk* inhibitors have been developed by pharmaceutical companies for treating metabolic disorders, with some notable success in recent clinical trials (*15*, *38*–*40*). While these drugs likely exert their actions through organs that express *Khk* (*41*), including the liver, intestines, kidneys, and adipose tissues, only the liver has garnered attention thus far (*42*). Our results suggest that the reported clinical benefits of these drugs are at least partly attributed to their actions on the small intestine. It will therefore be intriguing to measure dietary fat absorption in individuals with the loss-of-function mutations in *Khk* or individuals treated with *Khk* inhibitors.

Conversely, the appropriate absorption of dietary fat, especially essential fatty acids, is crucial not only for maintaining body weight but also for supporting organ health. Common intestinal pathologies, such as irritable bowel syndrome (*43*) or intestinal cancer (*44*), negatively influence intestinal functions, including fructose catabolism, through chronic inflammation and epithelial cell de-differentiation. While ∼20% of patients with cancer experience cancer-related cachexia, ∼60% of those with intestinal cancer suffer from this condition (*44*). However, no sufficient data are available regarding their fructose consumption or dietary fat absorption capacity. It will be necessary to investigate whether impaired dietary fat absorption contributes to cachexia progression in cancer patients who routinely consume fructose and whether intravenous fat provision may help mitigate cachexia (*45*).

Beyond these host-specific factors, our fecal transplant data reveal the importance of the gut microbiome in maintaining lacteal integrity, particularly in the ileum, under the condition of HFCS consumption. It is plausible to hypothesize that the gut microbiome plays different roles in different segments of the small intestine; however, this notion remains poorly understood. Only recently have studies begun to reveal that the microbiome in the ileum not only shows a closer resemblance to the overall gut microbiome than those in other small intestinal segments (*46*) but also reacts similarly to fructose feeding (*43*). Notably, FMT in NW-treated, non–antibiotic-treated recipient mice did not produce marked changes in body weight, glucose tolerance, or TG absorption. These findings suggest that, under normal physiological conditions without fructose intake and an intact resident gut microbiome, the effects of transferred microbiota may be limited. Thus, sustained HFCS exposure and/or prior depletion of the resident microbiota (e.g., exposure to antibiotics) would be necessary to allow this microbiome-dependent pathway to exert robust metabolic effects. Further studies will be necessary to understand how HFCS consumption rewires gut microbiome in an intestinal segment-specific manner, influencing lacteal integrity and dietary fat absorption.

In this regard, our findings that the gut microbiome affects the population of ileal macrophages under the condition of HFCS consumption present a previously unrecognized interaction between the host’s intestinal fructose catabolism and gut immune-microbe interactions. Our data highlight the potential contribution of Erysipelotrichaceae family to this process, and intriguingly, members of this family have been associated with diet-induced microbiome changes and lipid metabolism in obese mice (*31*–*33*, *47*). Functional studies on whether and how Erysipelotrichaceae family influences intestinal immune responses (e.g., activation, suppression, or immune cell recruitment) would help understand their potential role in lacteal growth and dietary fat absorption (*48*, *49*). Looking forward, identifying specific microbiome species that promote villus macrophage populations and lacteal growth will be instrumental in developing new probiotics to therapeutically modulate dietary fat absorption for the prevention and treatment of metabolic diseases.

## MATERIALS AND METHODS

### Mouse studies

Animal studies followed protocols approved by the Institutional Animal Care and Use Committee of the University of California, Irvine (no. AUP-25-122). Eight-week-old male C57BL/6 mice were purchased from Jackson Laboratory and group-housed (two to three mice per cage) on a standard light-dark cycle (7:00 to 19:00) with free access to chow (Teklad 2020X) and water. *Khk-C* floxed mice (*8*) were bred with *Villin*-Cre mice (stock no. 004586; Jackson Laboratory) to generate intestine-specific *Khk-C* knockout mice. For HFCS provision, mice were provided either ad libitum H_2_O or a 55% fructose:45% glucose mixture in their drinking water. Daily water and food intake were determined by measuring the total consumption in a cage and dividing by the number of mice in the cage. For antibiotics treatment, a cocktail of antibiotics, including 1 g liter^−1^ ampicillin, neomycin, metronidazole, and 0.5 g liter^−1^ vancomycin, was dissolved in HFCS water. For gut bacteria transplantation, fecal pellets were freshly collected from donor mice, immediately dissolved in phosphate-buffered saline (PBS; 2 ml), centrifuged at maximum speed at 4°C for 10 min, and the supernatant was transferred to antibiotics-treated recipient mice via oral gavage (200 μl per mouse) with a plastic feeding tube (Instech Laboratories) every 3 to 4 days for 8 weeks. Body composition was assessed using EchoMRI Whole Body Composition Analyzer (Houston, TX, United States), which provides measurements of whole body fat and lean mass.

### Glucose tolerance test

For glucose tolerance test, mice received d-glucose in sterile saline at a dosage of 2 g/kg via oral gavage after 12 hours of fasting. Glucose levels were determined from a tail snip with a hand-held glucometer (Accu-Check Guide) in the basal state and 15, 30, 60, and 120 min following glucose administration. For glucose and insulin measurements, serum was collected after 6 hours of fasting. Insulin levels were measured using an Ultra Sensitive Mouse Insulin ELISA Kit (catalog no. 90080; Crystal Chem). The HOMA-IR was calculated with the following formula (glucose levels in mM, insulin levels in uIU/ml): HOMA-IR = (glucose × insulin) / 22.5.

### Circulating hormone measurements

For circulating appetite and energy homeostasis-related hormone measurements, 10 μl of serum samples were centrifuged for 30 s to remove debris before being applied to a 384-well plate for hormone profiling. We used the MILLIPLEX MAP mouse MMHE-44K metabolic Hormone expanded panel kit (Millipore). All reagents were used at 10 μl to adjust the manufacturer’s protocol to our 384-well plate format. Outcomes from wells with values less than the detection limit for specific analytes were excluded from the study. Standards and quality controls were assayed in technical duplicates. Plates were washed in between incubations using a BioTek 406 Touch plate washer (BioTek). Samples were read using an xMAP INTELLIFLEX System (Luminex). Belysa Immunoassay Curve Fitting Software (Millipore) was used for curve fitting.

### Oral lipid absorption assay

For the dietary lipid absorption assay, mice received soybean oil via oral gavage (10 μl g^−1^) after 6 hours of fasting. Blood was collected via tail snip to measure circulating TG after different durations. Feces were snap-frozen in liquid nitrogen with a precooled Wollenberger clamp. Multiple cohorts were used, and data were combined if there was no statistical difference between cohorts. TG levels were measured using a TG colorimetric assay kit (catalog no. 10010303; Cayman Chemicals).

### Oxygen bomb calorimetry

To determine fecal energy loss, feces over the final 7-day period of experiments were weighed and measured for energy content (heat of combustion) using a 6400 isoperibol calorimeter with an 1138 oxygen bomb (Parr Instruments Co., Moline, IL, USA). Total fecal energy loss was calculated by multiplying cumulative fecal mass (gram) with energy density (kilocalorie/gram) to calculate total fecal energy loss (kilocalories).

### Immunofluorescence imaging

For whole-mount microscopy of intestinal segments, we adapted a previously established protocol (*50*). Intestinal samples were prepared carefully by removing the intestine from the mesentery of the pyloric ring with forceps. All samples were kept in a 15-cm, 6-well culture plate filled with ice-cold PBS. The extracted intestines were divided into three equal segments: proximal (duodenum), mid (jejunum), and distal (ileum), and feces and other luminal contents were flushed out using a syringe. The samples were longitudinally cut along the mesenteric border to expose the lumen and further sectioned into small pieces measuring 0.5 to 1 cm, then dissected into strips of intestinal villi. Tissue samples were fixed in 4% buffered paraformaldehyde overnight at 4°C. After fixation, tissues were sequentially dehydrated with 10% sucrose for 2 hours and 20% sucrose overnight at 4°C. Dehydrated tissues were subsequently frozen and embedded in Frozen Section Media (Leica) for cryosection and cut into 20-μm-thick sections using a Cryocut Microtome (Leica). Samples were then blocked in protein block serum (Agilent) with 0.3% Triton X-100 (Thermo Fisher Science) for 1 hour and incubated with primary antibodies in antibody diluent (Agilent) overnight at 4°C, followed by incubation with anti-mouse secondary antibodies for 2 hours at room temperature. Tissue sections were mounted with Vectashield plus DAPI (4′,6-diamidino-2-phenylindole) (Vector Labs) and imaged using an LSM 980 confocal microscope (Zeiss). The following primary antibodies were used: anti-CD31 (rat monoclonal, clone MEC 13.3, BD Bioscience; anti-F4/80 (rat monoclonal, clone BM8, BioLegend); anti–E-cadherin (goat polyclonal, R&D); and anti–LYVE-1 (rabbit polyclonal, AngioBio). Alexa 488-, Alexa 594-, and Alexa 647-conjugated secondary antibodies were purchased from Jackson ImmunoResearch. Morphometric analyses were conducted using data processed through LSM Zen (Carl Zeiss), ImageJ software (NIH), or Imaris (Bitplane). The blood capillary, lacteal, and epithelial lengths (villus heights) were measured for each villus. Given that lacteal length varies significantly but tends to correlate with villus or capillary length, we also calculated the ratios of lacteal to villus and lacteal to capillary lengths. These relative measurements are known to remain consistent along the entire length of the intestine (*51*). Each measurement represents the mean of six measurements per sample. Macrophage density was quantified by measuring the F4/80^+^ area across five 0.0265 mm^2^ fields per sample, with the results presented as relative values normalized to the area.

### Immunoblot

Liver tissues were prepared in RIPA [25 mM Tris-HCl (pH 7.6), 2 mM EDTA, 150 mM NaCl, 1% NP-40, 1% deoxycholate, and 0.1% SDS] lysis buffer supplemented with protease inhibitors [100 mM AEBSF, pepstatin A (1 μg/ml), leupeptin (10 μg/ml), and aprotinin (10 μg/ml)] and Pierce Phosphatase Inhibitor Mini Tablets. After 30 min on ice, lysed tissue was centrifuged at 13,000 rpm for 30 min at 4°C, and protein concentrations were quantified using a detergent-compatible protein assay (Bio-Rad). Lysates were boiled for 10 min at 95°C using Laemmli sample buffer to denature proteins. An equal amount of protein (10 to 45 μg) was resolved by SDS–polyacrylamide gel electrophoresis gels and proteins were transferred to nitrocellulose membrane (Amersham Biosciences). Membranes were blocked in Odyssey blocking buffer (LI-COR Biosciences) for 1 hour at room temperature, then incubated with primary antibodies: anti-rabbit pAkt (CST 4060), anti-rabbit Akt (CST 4691), anti-rabbit β-Actin (CST 4970S), and IRDye-conjugated secondary antibodies. Immunoblot signals were visualized and quantified using the Odyssey imaging system (LI-COR Biosciences).

### Histology

For H&E staining, freshly collected intestinal tissues were fixed in 4% paraformaldehyde overnight, embedded in paraffin, sectioned, and stained with H&E. Tissues were submitted to the Experimental Tissue Shared Resource Facility at the University of California Irvine. Images were captured using a high-resolution image scanner (Ventana DP200 ROCHE). For Oil Red O staining, freshly collected hepatic tissues were fixed in 4% paraformaldehyde overnight, dehydrated by titrating in sucrose (10 and 20%) and embedded in Frozen Section Media (Leica) for cryosection using a Cryocut Microtome (Leica), and stained with Oil Red O. Images were captured using a Nano-Zoomer Digital Pathology scanner (Hamamatsu, Japan).

### RT-qPCR

RNA samples were prepared using TRIzol Reagent (Invitrogen) according to the manufacturer’s instructions. RNA was reverse transcribed to cDNA using the iScript kit (Bio-rad). The resulting cDNA was analyzed by quantitative reverse transcription polymerase chain reaction (qPCR) using SYBR green master mix (Life Technologies) on QuantStudio6 Real-Time PCR system (Life Technologies). Relative mRNA expression was calculated from the comparative threshold cycle (C_t_) values relative to housekeeping genes *Actin*, *36b4*, and *Tbp*. Primer sequences for RT-qPCR are: *Glut5* (forward: TCTTTGTGGTAGAGCTTTGGG, reverse: GACAATGACACAGACAATGCTG), *Khk-a* (forward: TTGCCGATTTTGTCCTGGAT, reverse: CCTCGGTCTGAAGGACCACAT), *Khk-c* (forward: TGGCAGAGCCAGGGAGAT, reverse: ATCTGGCAGGTTCGTGTCGTA), *Aldob* (forward: CACCGATTTCCAGCCCTC, reverse: GTTCTCCACCTTTATCCTTTGC), *Acly* (forward: CAGCCAAGGCAATTTCAGAGC, reverse: CTCGACGTTTGATTAACTGGTCT), *Acss2* (forward: ATGGGCGGAATGGTCTCTTTC, reverse: TGGGGACCTTGTCTTCATCAT), *Fasn* (forward: GGAGGTGGTGATAGCCGGTAT, reverse: TGGGTAATCCATAGAGCCCAG), *Scd1* (forward: TTCAGAAACACATGCTGATCCTCATAATTCCC, reverse: ATTAAGCACCACAGCATATCGCAAGAAAGT), *Tbp* (forward: CCCTATCACTCCTGCCACACCAGC, reverse: GTGCAATGGTCTTTAGGTCAAGTTTAC), *Actin* (forward: CCCTGTATGCTCTGGTCGTACCAC, reverse: GCCAGCCAGGTCCAGACGCAGGATG), and *36b4* (forward: GGAGCCAGCGAGGCCACACTGCTG, reverse: CTGGCCACGTTGCGGACACCCTCC).

### 16*S* rRNA gene amplicon sequencing and analysis

Fresh feces samples were collected at 9 a.m., snap-frozen, and stored at −80°C for 16*S* rRNA sequencing. The fecal DNA was extracted using the ZymoBIOMICS 96 DNA Kit (Zymo Research) or the Qiagen DNeasy UltraClean Microbial Kit (Qiagen 12224-50). 16*S* rRNA amplicon PCR was performed targeting the V4-V5 region using the EMP primers [515F (barcoded) and 926R] (*52*, *53*). The sequencing library was prepared using an innovative library preparation process, where PCR reactions were performed in real-time PCR machines to control cycles and thus limit PCR chimera formation. The library was sequenced on Illumina MiSeq version 3 chemistry with a PE300 sequencing length. Unique amplicon sequence variants were inferred from raw reads using the DADA2 pipeline. The sequences were assigned a taxonomic classification using the August 2013 greengenes database (https://greengenes.lbl.gov/), trained with the primer pairs that were used to amplify the 16*S* region. The extracted region was truncated at 243 bp for single read analysis which should give the best taxonomical identification. Composition visualization, alpha-diversity, and beta-diversity analyses were performed using Qiime version 2. Taxa with differential abundance and prevalence between the WT and *Khk-C*^∆Villi^ groups were identified by running MaAsLin3 (*54*) using parameters --normalization = “TSS” --transform = “LOG” --fixed_effects = (“*Khk-C*^∆Villi^.gene”) --reference = (“*Khk-C*^∆Villi^.gene, WT”) on samples from days 14 to 84. *Allobaculum* spp. was the only genus with significant differential abundance between the WT and *Khk-C*^∆Villi^ groups at a false discovery rate threshold of 0.05, and no taxa had significantly different prevalence at this threshold. For the diet switching cohort, we amplified the V4 region of the 16*S* rRNA gene by using CUPID-seq (combinatorial, unique, phased, in-line dual-indexed sequencing) to add two rounds of unique dual indexes before sequencing (*55*). The libraries were sequenced on the Pacific Biosciences AVITI platform with a PE300 sequencing length and demultiplexed using a custom pipeline. Unique amplicon sequence variants were inferred from raw reads using the DADA2 pipeline (*56*). The sequences were assigned a taxonomic classification using the Silva version 138 database (*57*). Composition visualization, alpha-diversity, and beta-diversity analyses were performed using a combination of custom scripts and phyloseq (*58*). Taxa in the WT mice with differential abundance from the NW treatment were identified by running MaAsLin3 (*54*) using parameters --normalization = “TSS” --transform = “LOG” --formula=“∼ Treatment + numReads”, reference = c(“Treatment,NW”). Customed scripts and phyloseq to analyze 16*S* rRNA-seq are publicly available (*58*). Sequence data are provided as table S1.

### Statistical analysis

Otherwise indicated, a two-tailed, unpaired Student’s *t* test was used to calculate *P* values, with *P* < 0.05 used to determine statistical significance. One-way analysis of variance (ANOVA) followed by Tukey’s test was used for multiple comparisons. Outliers were determined by 1.5 times the interquartile range (1.5 * IQR below Q1 or 1.5 * IQR above Q3) (*59*, *60*). Sample sizes were determined by power analysis based on preliminary data obtained in the lab. Blinded quantification and randomization was performed to avoid bias.
