# Supplementary material for: Intestinal fructose catabolism promotes obesity and insulin resistance via ileal lacteal remodeling
Source: Sci Adv. 2026 Aug 28;12(35):eaec0481. doi: 10.1126/sciadv.aec0481 (PMC13524015; doi:10.1126/sciadv.aec0481)
Supplement: Supplementary file 1 [file sciadv.aec0481_sm.pdf]

Science Advances
AAAS

Supplementary Materials for

Intestinal fructose catabolism promotes obesity and insulin resistance via ileal lacteal remodeling

Miranda L. Lopez *et al.*

Corresponding author: Hosung Bae, hosungb@hs.uci.edu; Cholsoon Jang, choljang@uci.edu

Sci. Adv. **12**, eaec0481 (2026)
DOI: 10.1126/sciadv.aec0481

The PDF file includes:

Figs. S1 to S7
Legend for table S1

Other Supplementary Material for this manuscript includes the following:

Table S1

Figure S1

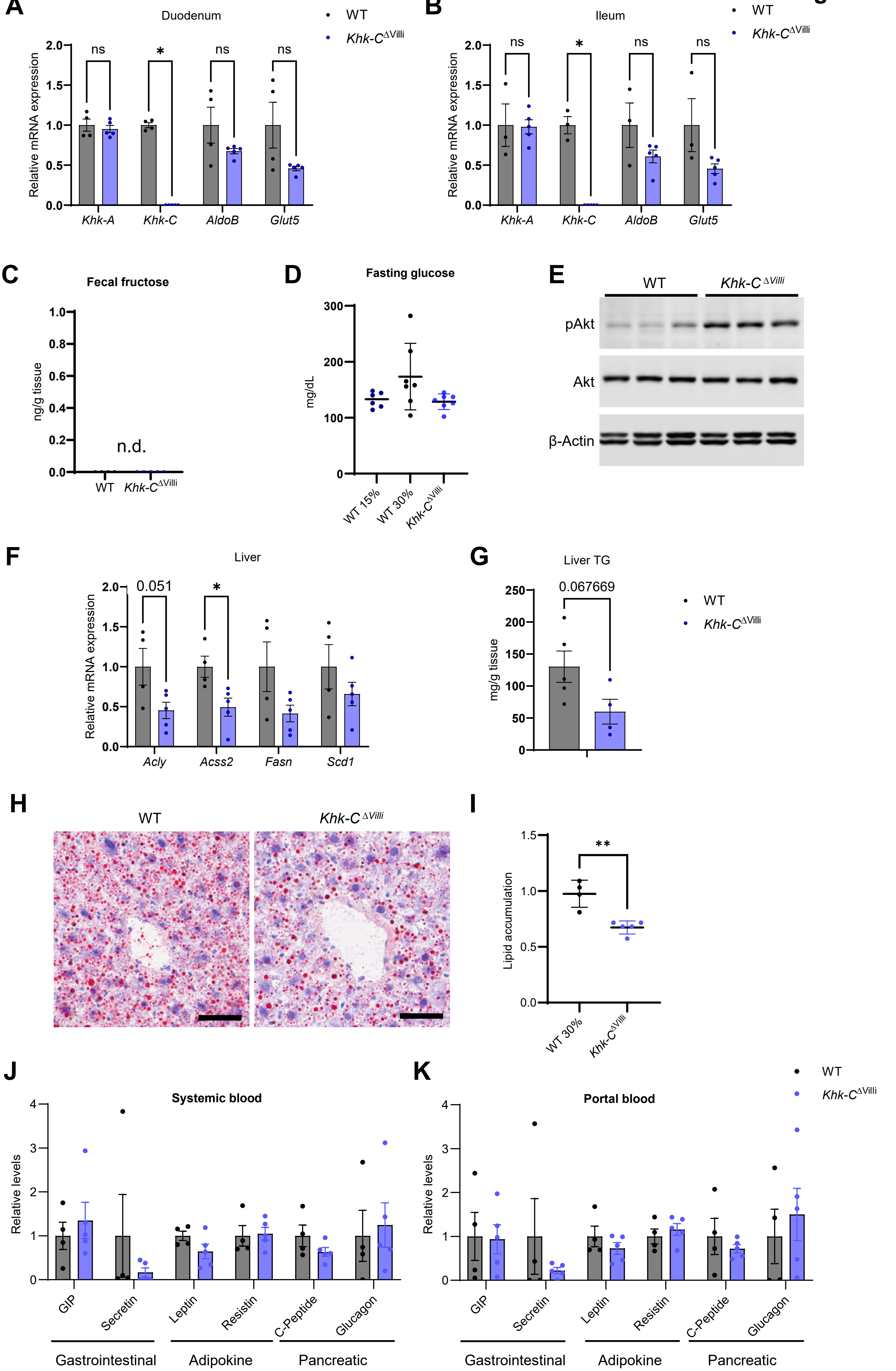

A
Duodenum
WT
Khk-C^ΔVilli
Relative mRNA expression
ns
*
ns
ns
Khk-A
Khk-C
AldoB
Glut5
B
Ileum
WT
Khk-C^ΔVilli
Relative mRNA expression
ns
*
ns
ns
Khk-A
Khk-C
AldoB
Glut5
C
Fecal fructose
ng/g tissue
n.d.
WT
Khk-C^ΔVilli
D
Fasting glucose
mg/dL
WT 15%
WT 30%
Khk-C^ΔVilli
E
WT
Khk-C ΔVilli
pAkt
Akt
β-Actin
F
Liver
Relative mRNA expression
0.051
*
Acly
Acss2
Fasn
Scd1
G
Liver TG
0.067669
mg/g tissue
WT
Khk-C^ΔVilli
H
WT
Khk-C ΔVilli
I
Lipid accumulation
**
WT 30%
Khk-C^ΔVilli
J
Systemic blood
Relative levels
GIP
Secretin
Leptin
Resistin
C-Peptide
Glucagon
Gastrointestinal
Adipokine
Pancreatic
K
Portal blood
WT
Khk-C^ΔVilli
Relative levels
GIP
Secretin
Leptin
Resistin
C-Peptide
Glucagon
Gastrointestinal
Adipokine
Pancreatic

Supplementary Fig 1. Suppression of intestinal fructose does not affect body weight under normal water conditions. (A, B) Fructose catabolism gene expression in duodenum and ileum (n_{WT} = 4, $n_{Khk\text{-}C}$ = 5). **(C)** Measurement of fecal fructose (n_{WT} = 4, $n_{Khk\text{-}C}$ = 5). **(D)** Fasting (6hr) glucose levels ($n_{WT15\%}$ = 6, $n_{WT30\%,\ Khk\text{-}C}$ = 7). (**E**) Immunoblot analysis of hepatic insulin sensitivity. (**F**) De novo lipogenesis gene expression in liver (n_{WT} = 4, $n_{Khk\text{-}C}$ = 5). (**G**) Liver TG levels (n_{WT} = 4, $n_{Khk\text{-}C}$ = 5). (**H, I**) Representative liver Oil Red O staining and quantitation of lipid accumulation. Staining was performed in 4~5 mice per group, and lipid accumulation was quantified in four randomly selected areas per liver. Scale bars, 20 µm. **(J, K)** Systemic and portal blood levels of hormones (n_{WT} = 4, $n_{Khk\text{-}C}$ = 5). Data are mean±s.d. ns, not significant. *$p<0.05$ by Student's t-test.

Figure S2

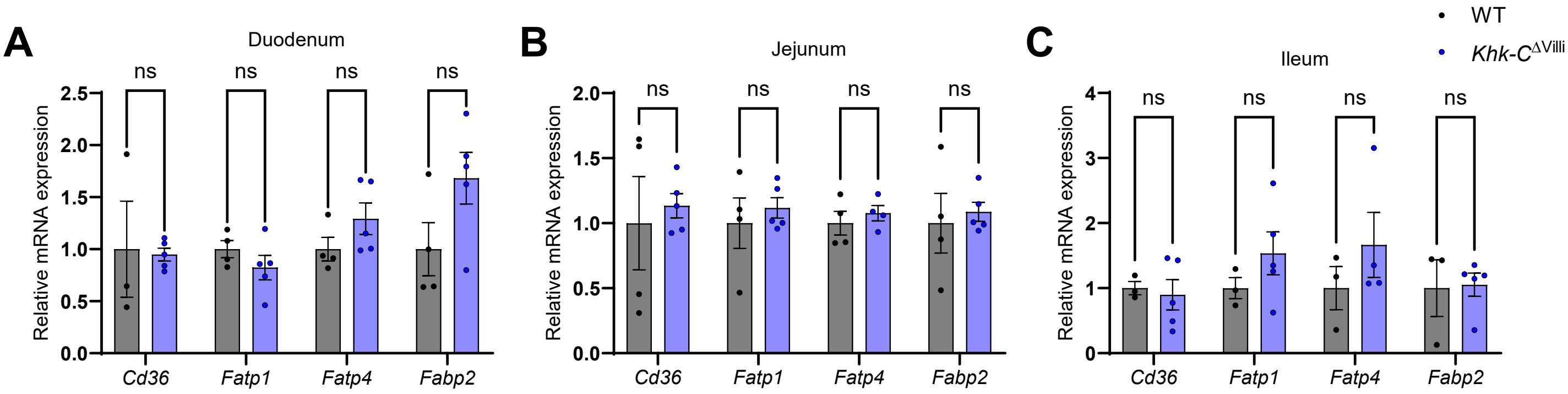

Supplementary Fig 2. Suppression of intestinal fructose catabolism does not alter fatty acid transporter expression under HFCS consumption. (A-C) Relative fatty acid transporter gene expression in duodenum, jejunum, and ileum (n_{WT} = 4, $n_{Khk\text{-}C}$ = 5). Data are mean±s.d. ns, not significant.

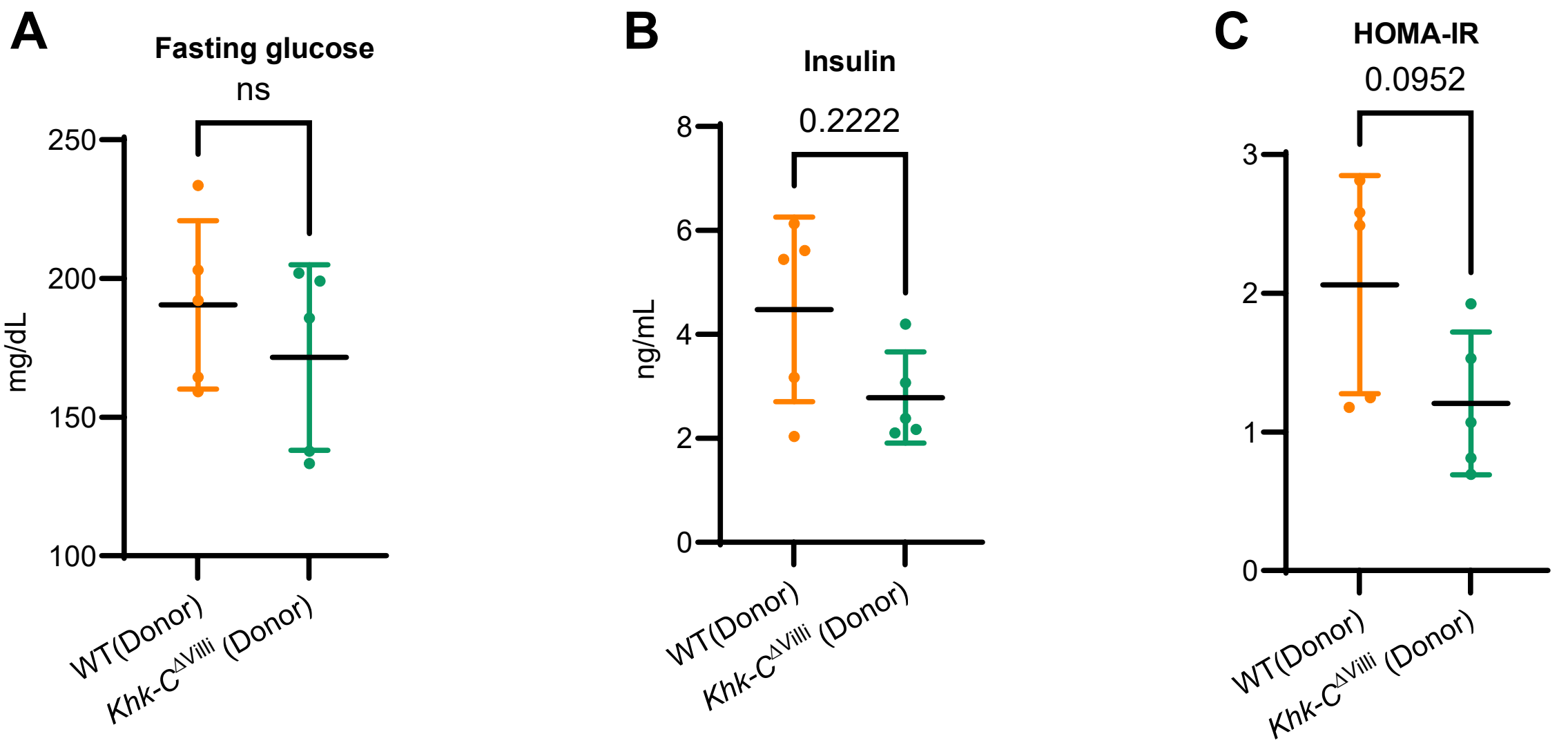

Supplementary Fig 3. Glucose and insulin sensitivity after fecal microbiome transplant
Fasting glucose **(A)**, insulin **(B)**, homeostatic model assessment of insulin resistance (HOMA-IR) **(C)** ($n_{WT(Donor)}$ = 5, $n_{Khk\text{-}C(Donor)}$ = 5). Data are mean±s.d. ns, not significant.

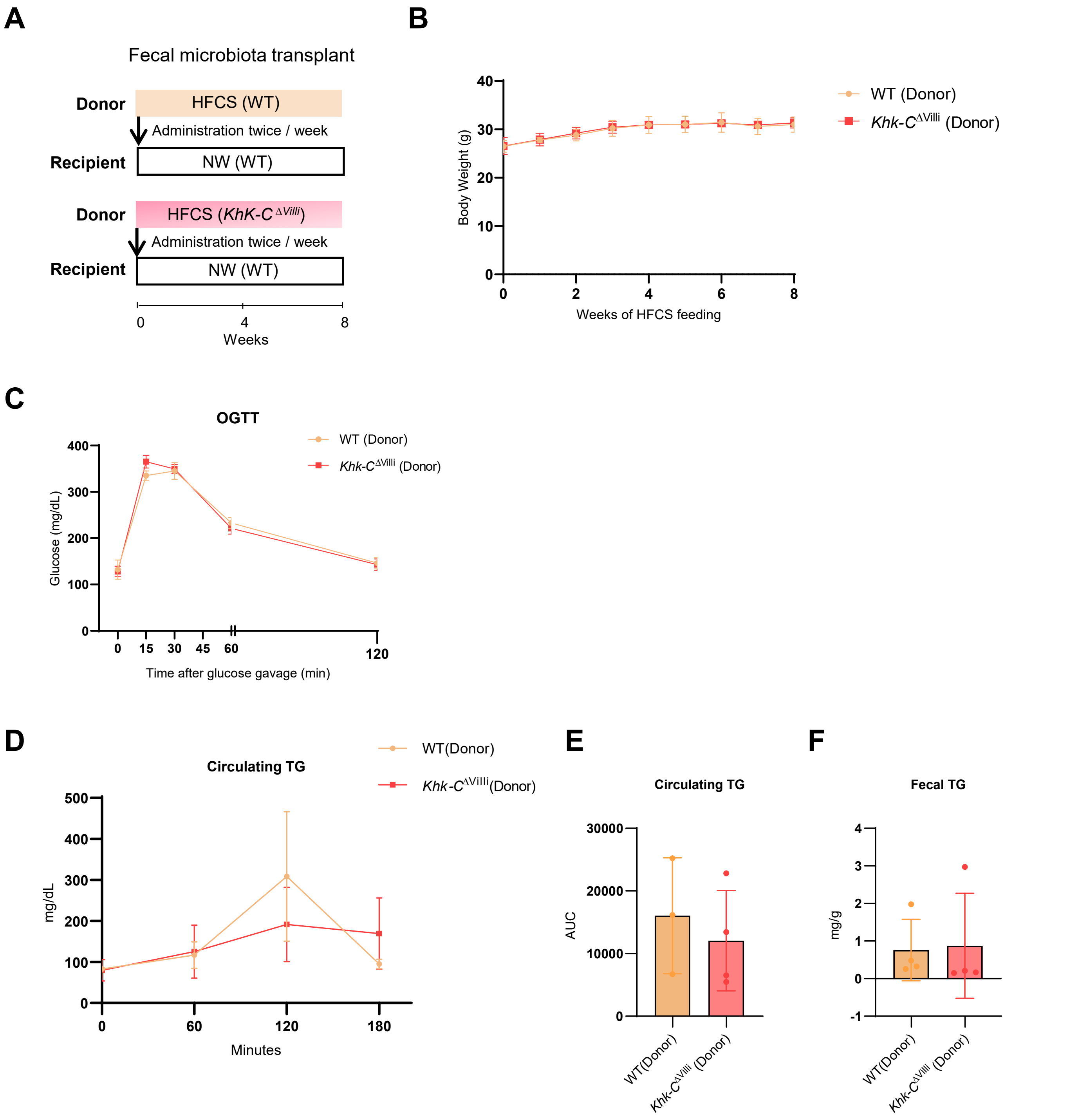

Supplementary Fig 4. Prior gut microbiome depletion and sustained HFCS exposure are required for fecal transplant to phenocopy donor-derived traits.
(A) Schematic of fecal transplant (twice/week) from donors (WT or *Khk-C* $^{\Delta Villi}$ mice on HFCS for 12 weeks; n = 4) to recipients (WT without antibiotics; n = 4) with NW drinking for 8 weeks. **(B)** Body weight gain of the recipient mice after 8 weeks of fecal transplant and NW drinking. **(C)** OGTT. **(D-F)** Measurements of circulating TG levels (**D**) and the AUC (**E**), and fecal TG levels (**F**) in the recipient mice following soybean oil gavage.

Figure S5

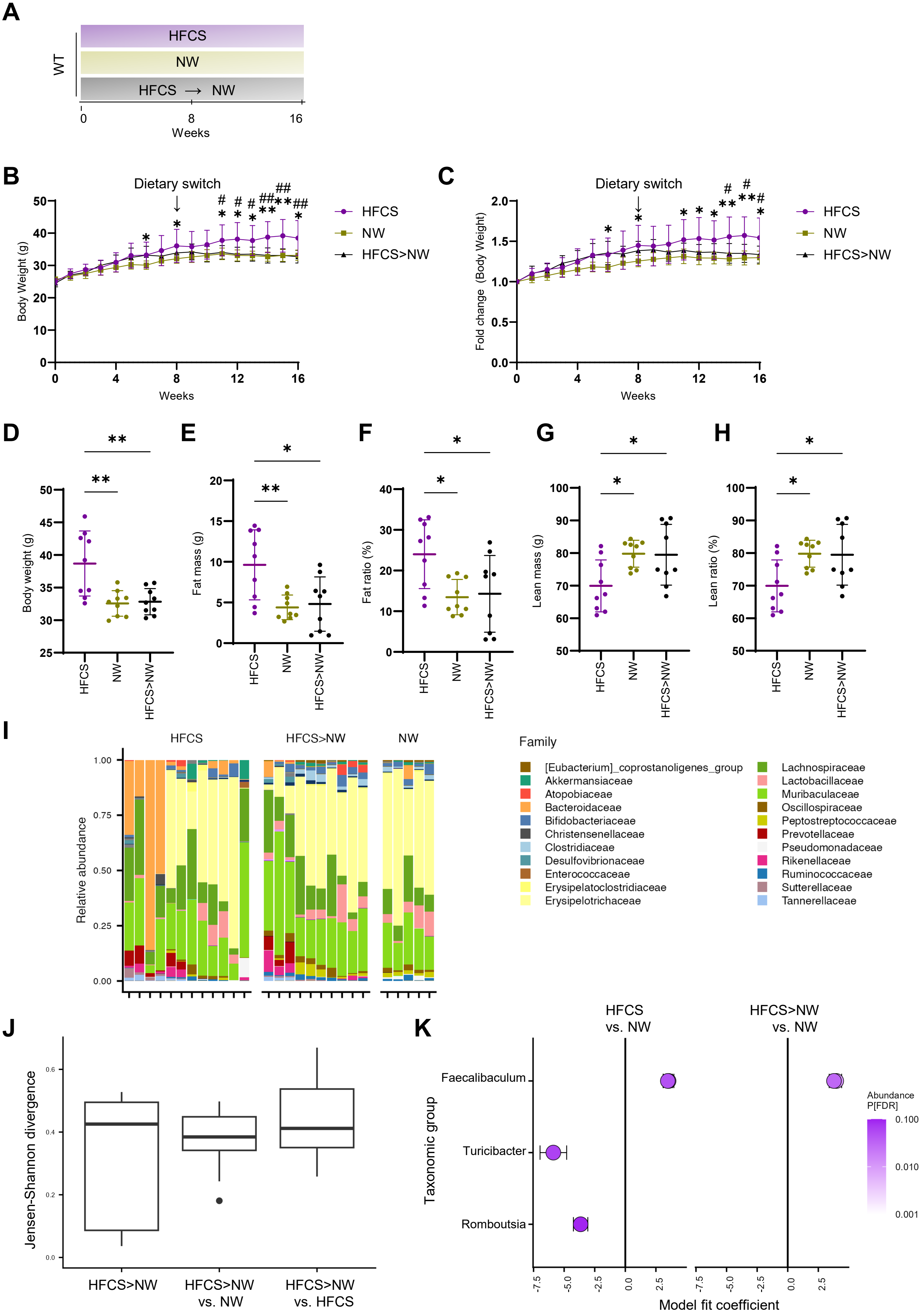
A
WT
HFCS
NW
HFCS → NW
0
8
16
Weeks
B
Dietary switch
Body Weight (g)
Weeks
HFCS
NW
HFCS>NW
C
Dietary switch
Fold change (Body Weight)
Weeks
HFCS
NW
HFCS>NW
D
Body weight (g)
E
Fat mass (g)
F
Fat ratio (%)
G
Lean mass (g)
H
Lean ratio (%)
HFCS
NW
HFCS>NW
I
HFCS
HFCS>NW
NW
Relative abundance
Family
[Eubacterium]_coprostanoligenes_group
Akkermansiaceae
Atopobiaceae
Bacteroidaceae
Bifidobacteriaceae
Christensenellaceae
Clostridiaceae
Desulfovibrionaceae
Enterococcaceae
Erysipelatoclostridiaceae
Erysipelotrichaceae
Lachnospiraceae
Lactobacillaceae
Muribaculaceae
Oscillospiraceae
Peptostreptococcaceae
Prevotellaceae
Pseudomonadaceae
Rikenellaceae
Ruminococcaceae
Sutterellaceae
Tannerellaceae
J
Jensen-Shannon divergence
HFCS>NW
HFCS>NW vs. NW
HFCS>NW vs. HFCS
K
HFCS vs. NW
HFCS>NW vs. NW
Taxonomic group
Faecalibaculum
Turicibacter
Romboutsia
Model fit coefficient
Abundance P[FDR]
0.100
0.010
0.001

Supplementary Fig 5. Switching from HFCS to NW restores body weight and body composition. (A) Schematic of HFCS, NW, and dietary switch group HFCS>NW (8 weeks each) for 16 weeks (n = 9 each). **(B, C)** Body weight changes during 16 weeks of dietary regimen. *$p<0.05$, **$p<0.01$ (HFCS vs. NW) or $^{\#\#}p<0.001$ $^{\#\#}p<0.001$ (HFCS vs. HFCS>NW) by Student's t-test. **(D-H)** Final body weight, fat mass and the ratio of fat mass to body weight (%), lean mass and the ratio of lean mass to body weight (%). *$p<0.05$, **$p<0.01$ by one-way ANOVA followed by Tukey's test. **(I)** Representative bacterial family compositions from each group. **(J)** Jensen-Shannon divergence between mice that had undergone a diet switch (HFCS-NW) and the mice that had consumed either NW or HFCS alone. **(K)** Differential abundance results from MaAsLin3 showing the five taxa with the strongest associations between indicated groups. X-axis represents the model fit coefficient (β) from MaAsLin3, indicating the direction and magnitude of association with treatment groups.

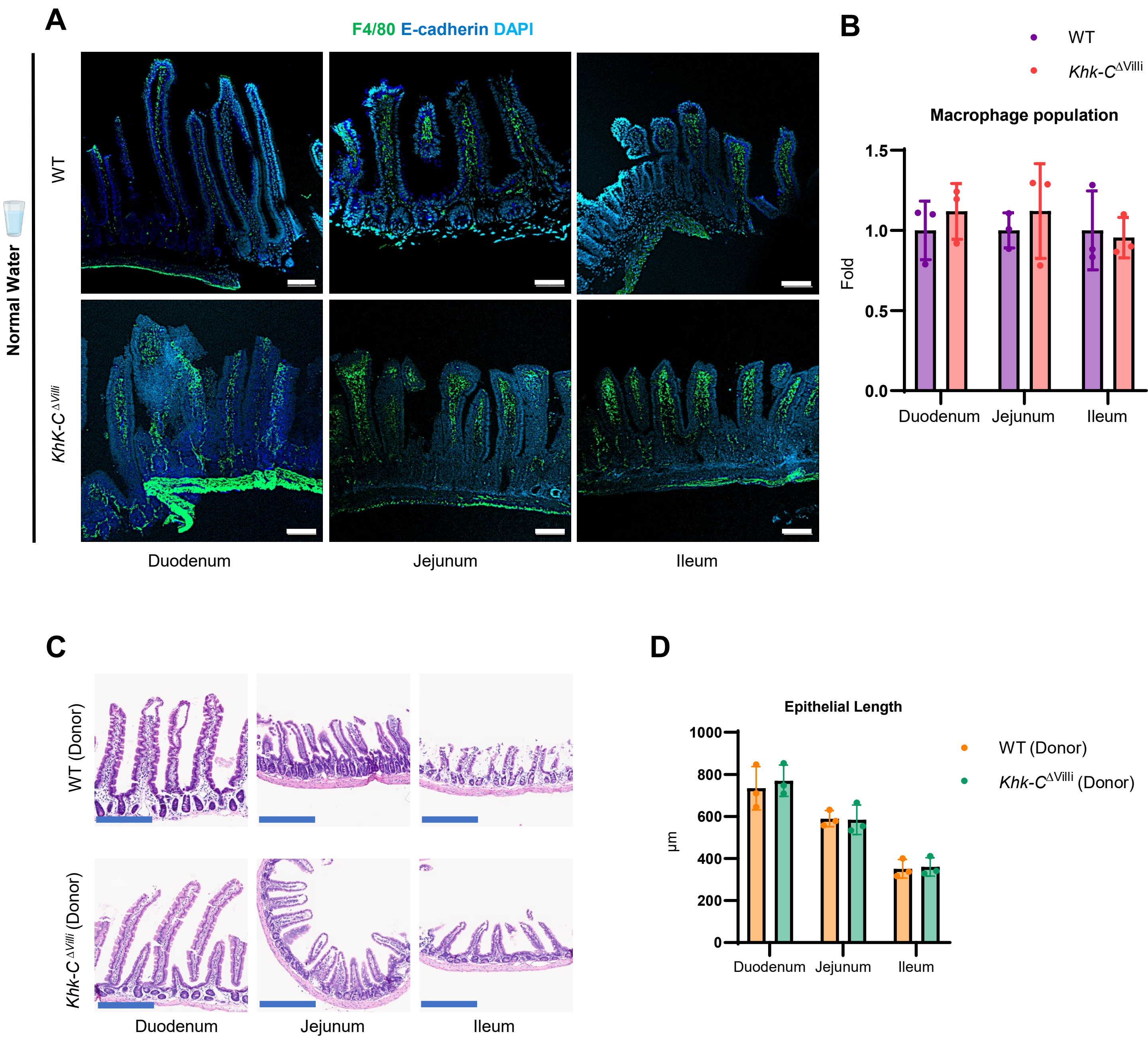

Supplementary Fig 6. Suppression of intestinal fructose catabolism does not reduce villus macrophage population under normal water conditions. (A) Representative immunofluorescence images of intestinal cryosections showing F4/80-positive macrophages (green), E-cadherin-positive epithelial cells (blue), and nuclei (DAPI, turquoise). Scale bars, 100 μm. **(B)** Relative abundance of macrophages in mice on normal water for 12 weeks (n = 3). Data are mean±s.d. **(C)** Representative H&E images of intestinal tissues in the recipient mice after 8 weeks of fecal transplant and HFCS drinking. Scale bars, 200 μm. **(D)** Epithelial length of intestinal tissues in the recipient mice after 8 weeks of fecal transplant and HFCS drinking (n = 3). Data are mean±s.d.

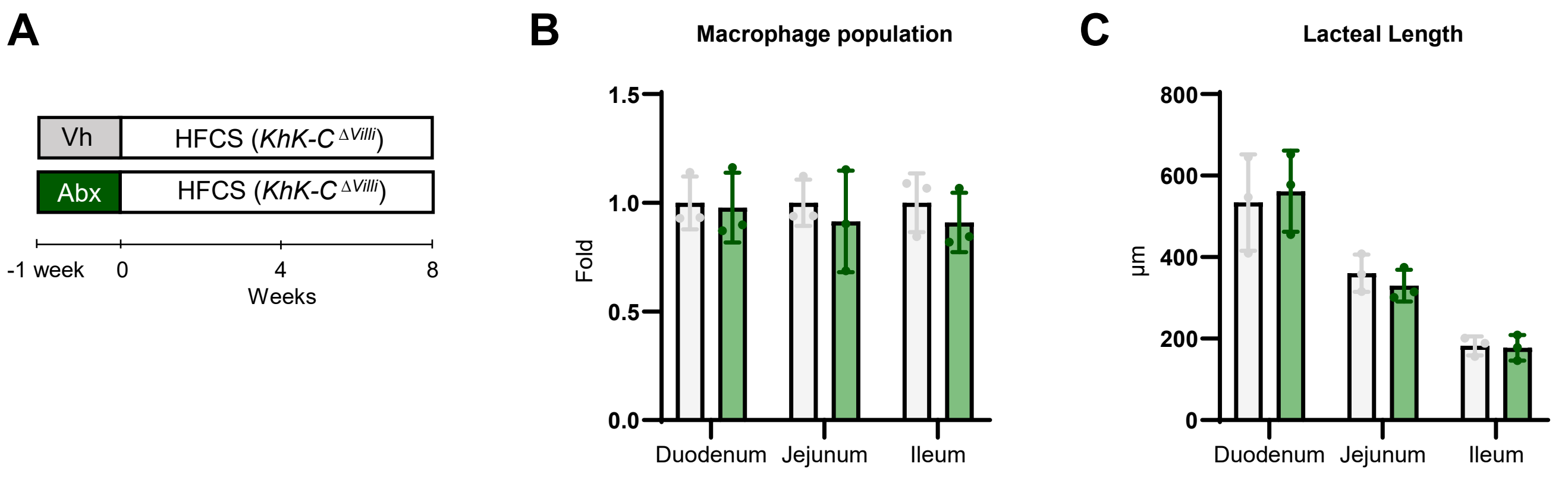

Supplementary Fig 7. Antibiotics treatment does not affect macrophages and lacteal length in *Khk-C*$^{\Delta Villi}$ mice. **(A)** 8-week-old *Khk-C*$^{\Delta Villi}$ male mice were pretreated with either vehicle or antibiotics prior to 8 weeks of 30% HFCS administration. **(B, C)** Relative abundance of macrophages and lacteal length (n = 3). Data are mean±s.d.

Table S1. 16S rRNA sequencing analysis of WT and *Khk-C* $^{\Delta Villi}$ mice over a 12-week HFCS feeding period, assessed at multiple time points (0, 1, 3, 7, 14, 28, and 84 days).
